# The formation and function of ER-endosome membrane contact sites^☆^

**DOI:** 10.1016/j.bbalip.2016.01.020

**Published:** 2016-08

**Authors:** Emily R. Eden

**Affiliations:** UCL Institute of Ophthalmology, London, UK

**Keywords:** Endosome, Endoplasmic reticulum, Membrane contact site, Lipid transport

## Abstract

Recent advances in membrane contact site (MCS) biology have revealed key roles for MCSs in inter-organellar exchange, the importance of which is becoming increasingly apparent. Roles for MCSs in many essential physiological processes including lipid transfer, calcium exchange, receptor tyrosine kinase signalling, lipid droplet formation, autophagosome formation, organelle dynamics and neurite outgrowth have been reported. The ER forms an extensive and dynamic network of MCSs with a diverse range of functionally distinct organelles. MCSs between the ER and endocytic pathway are particularly abundant, suggesting important physiological roles. Here, our current knowledge of the formation and function of ER contact sites with endocytic organelles from studies in mammalian systems is reviewed. Their relatively poorly defined molecular composition and recently identified functions are discussed. In addition, likely, but yet to be established, roles for these contacts in lipid transfer and calcium signalling are considered. This article is part of a Special Issue entitled: The cellular lipid landscape edited by Tim P. Levine and Anant K. Menon.

## Highlights

• ER protein VAPA interacts with endosomal proteins at membrane contact sites (MCS).
• MCS function in endosome fission, positioning and growth factor receptor regulations.
• Proposed roles for ER-endosome MCS in lipid transport and Ca^2 +^ signalling

## 1 Introduction

**Fig. 1:**
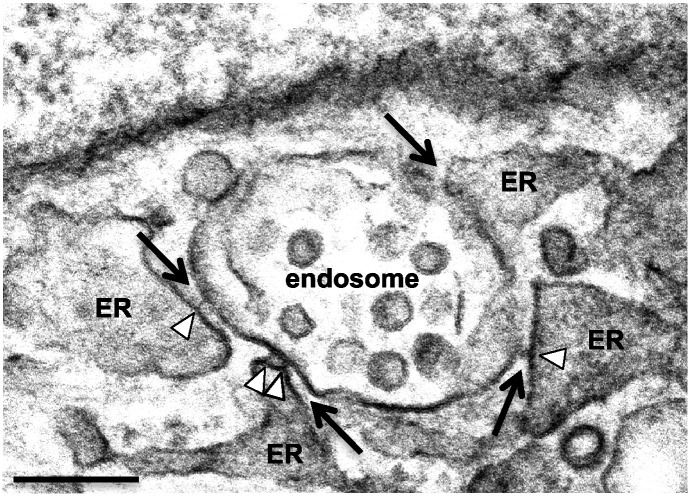
Electron micrograph of an ER-endosome membrane contact site. Hela cells were prepared for transmission electron microscopy. The image shows membrane contact sites (black arrows) between the ER and an endosome. Tethers (white arrowheads) between the two organelles are often visible at the contact site. Scale bar, 200 nm.

Membrane contact sites (MCSs) are regions of close apposition (≤ 30 nm) between two organelles, establishing microdomains for interorganellar exchange. The ER network forms extensive MCSs with multiple organelles within the cell, including the plasma membrane, mitochondria and Golgi apparatus. The first indication that the ER might make functional contact sites with endocytic organelles came from studies in yeast that identified a junction between the nucleus and vacuole formed by direct interaction between the ER protein Nvj1 and the vacuole protein Vac8 [1]. Although extremely abundant, ER contacts with the endocytic pathway (Fig. 1) in mammalian cells were only recently described [2], [3]. Membrane contact sites are stabilized by tethering complexes that maintain close proximity between the apposing membranes without membrane fusion. These tethers can often be discerned by electron microscopy, as multiple strands between the apposing membranes of the two organelles (Fig. 1). While our understanding of the composition of tethering complexes remains incomplete, with recent advances in membrane contact site biology, many organelle-specific tethering proteins have been identified [4], [5], [6]. The ER-localized Vesicle associated membrane protein (VAMP)-Associated Proteins (VAPs) have been implicated in tethering numerous different MCSs. For example, ER:mitochondrial MCSs are promoted by VAPB:PTPIP51 interaction [7],while ER:Golgi MCSs are promoted by VAP interaction with OSBP, CERT and Nir2 FFAT (diphenylalanine in an acidic tract) motifs [8]. A VAP homologue in yeast (Scs2) has been implicated in MCSs between the ER and the plasma membrane that are important in the regulation of phosphoinositide lipid turnover [9]. Moreover, VAP interaction with the FFAT motifs of the sterol binding proteins ORP1L [3] and STARD3 [10] on late endosomes/lysosomes have been implicated in MCS formation between the ER and the endocytic pathway, as will be discussed in more detail below.

## 2 ER-endosome contact site formation

**Fig. 2:**
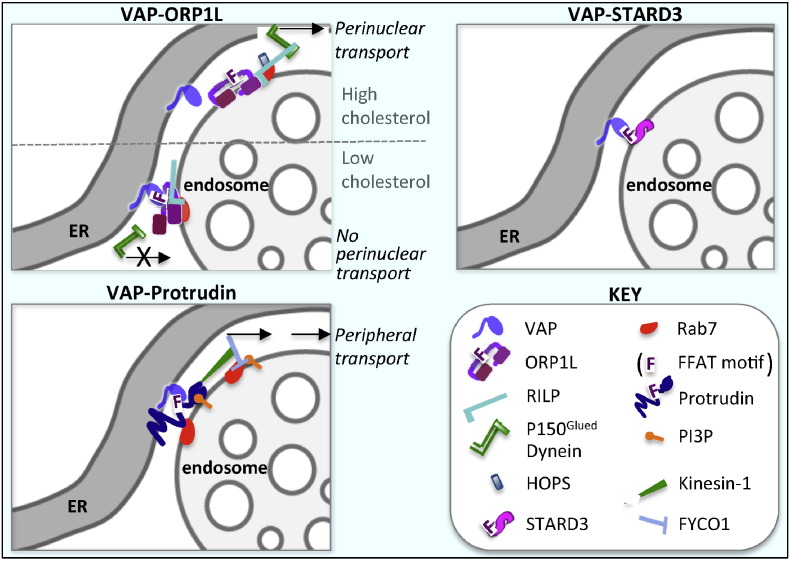
VAP interactions with FFAT motif-containing proteins at ER-endosome contact sites. VAP interacts with the FFAT motif of ORP1L when LDL-cholesterol is low, terminating ORP1L/RILP/dynein-mediated minus-end directed transport towards the perinuclear compartment. VAP also interacts with the FFAT motif of STARD3, another endosomal sterol-binding protein, promoting contact site formation. VAP additionally binds the FFAT motif of an ER-anchored protein, protrudin, which, through coincident binding of Rab7 and PI3P, mediates kinesin1-dependent, plus-end directed, transport towards the periphery of the cell.

The regulation and molecular composition of ER-endocytic organelle MCSs remains poorly characterized, but three independent studies implicate VAPs in tethering these contacts (Fig. 2), either by direct interaction with the FFAT motifs of endosomal sterol-binding proteins ORP1L and STARD3, or indirectly, by interaction with another integral ER membrane protein, protrudin, which interacts with Rab7 and phosphatidylinositol 3-phosphate (PI3P) at the endosome [11].

ORP1L is a sterol and oxysterol binding protein [12] that is recruited to a population of endosomes that are positive for the Neimann-Pick type C (NPC) protein NPC1^13^ by interaction with Rab7 [14]. Under conditions of low cholesterol in the endocytic pathway, ORP1L undergoes a conformational change that promotes VAP interaction [3]. Overexpression of an ORP1L mutant (ΔORD) in which the c-terminal oxysterol-binding domain had been removed to mimic ORP1L conformation under cholesterol-free conditions (thereby favouring VAP interaction) increased both the number and size of ER-endosome contact sites [3]. Similarly, over-expression of STARD3 or STARD3NL, which localize to a different population of earlier endosomes that contain the cholesterol transporter ABCA3 [13], resulted in a striking extension of ER-endosome contacts [10]. These data signal a role for VAP interactions with endosomal sterol-binding proteins in the formation of these contact sites, but it should be noted that while the increase in contact sites mediated by STARD3 overexpression was VAP-dependent, neither study reported a reduction in basal contact site formation on depletion of VAP/ORP1L/STARD3. It is therefore possible that VAP interactions might function at and/or stabilize existing ER-endosome contacts rather than initiating contact site formation.

As mentioned above, in addition to the interactions with endosomal FFAT-motif-containing proteins, VAPA also binds the FFAT motif of an integral ER membrane protein, protrudin [11]. Protrudin also contains a PI3P-interacting FYVE domain and a low complexity region through which it interacts with Rab7. Coincident interaction of ER-localized protrudin with endosomal PI3P and RAB7 promotes the formation of ER-endosome contact sites and the recruitment of VAPA to the contact [11]. Overexpression of protrudin results in rearrangement of the ER to form multiple and extended contacts, demonstrating a role in recruiting the ER to the contact site, but whether protrudin stabilizes pre-existing contacts or initiates contact site formation remains unclear.

**Fig. 3:**
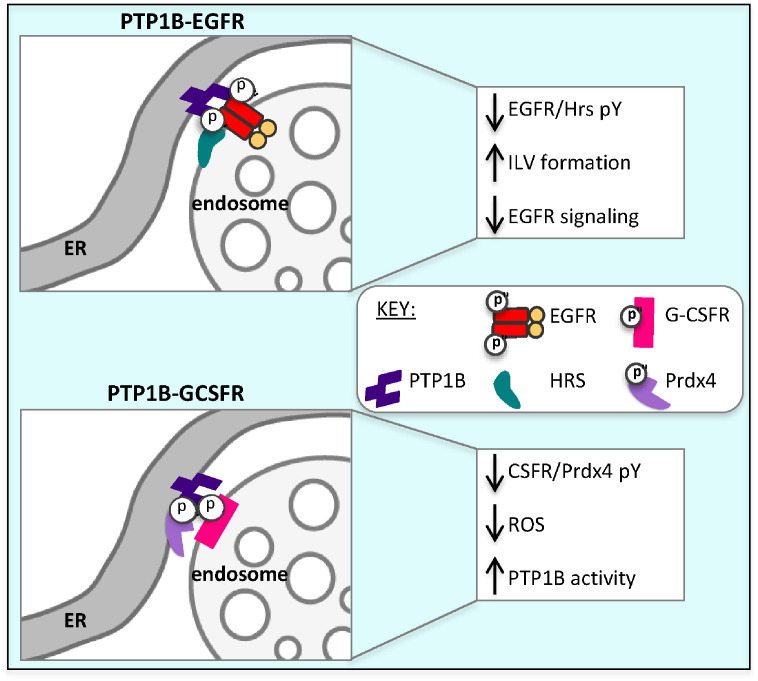
PTP1B interactions at ER-endosome contact sites. PTP1B dephosphorylates both endocytosed EGFR and HRS, promoting ILV formation and downregulation of EGFR tyrosine kinase activity. PTP1B also dephosphorylates both G-CSFR and Prdx4.

Another interaction reported to stabilize ER-endosome contact sites is that between the ER localized protein tyrosine phosphatase 1B (PTP1B) with endocytosed EGFR (Fig. 3). Depletion of PTP1B reduced contact sites and EGF stimulation increased the size of contacts, but these contact sites still formed in the absence of PTP1B or EGF [2], suggesting that existing contact sites provide platforms for PTP1B-EGFR interactions, which stabilize what would otherwise be extremely transient sites of contact. As yet there is no evidence for recruitment of VAPs to these contact sites, but as key regulators are identified, a role for VAPs might also become apparent.

Interestingly, in addition to binding Rab7, ORP1L, like protrudin, also binds phosphoinositides, through its PH domain [14], but the significance of this in its role at contact sites has not been established. Rab7 interactions are required for both protrudin- and ORP1L-regulated ER-endosome contact sites, suggesting a central role for this small GTPase in the regulation of ER-endosome contact sites. A Rab exchange from Rab5 to Rab7 marks the maturation of an early, sorting endosome, to a late endosome [15]. That PTP1B-EGFR interactions occur at early (Rab5-positive) as well as mature (Rab7-positive) endosomes, suggests that the regulation of ER-endosomes MCSs might involve several key tethering complexes specific to different endosome populations, possibly defined by the Rab5–Rab7 switch.

## 3 Functions of ER-endosome contact sites

### 3.1 Endosome fission and positioning

Endocytic organelles are extremely dynamic, continuously moving as they progress from initial endocytosed vesicles at the plasma membrane to sites of fusion with the predominantly perinuclear lysosomal compartment. However, not all cargo is destined for lysosomal degradation and many sorting, fusion and fission events occur along the pathway mediating alternative fates, notably recycling to the plasma membrane or retrograde transport to the trans-Golgi network [16]. ER-endosome contact sites were recently shown to define the position and timing for fission events that mediate the budding away of a tubular Rab4-positive transferrin-containing compartment to be recycled to the cell surface from a vacuolar Rab5-positive EGFR-containing early endosome that is destined for lysosomal fusion [17]. Sorting events continue to occur on late, Rab7-positive endosomes, with the retromer complex sorting cargo into specialized domains for retrograde transport to the Golgi [18]. FAM21 localizes to these sorting domains and ER tubules were found to make endosome contact sites at FAM21-positive sorting domains immediately prior to fission. EGF is again retained in the vacuolar, Rab7-positive compartment, while a smaller compartment, also positive for Rab7, buds away [17].

Endosomes destined for lysosomal fusion are trafficked along the cytoskeleton as they undergo sorting, fusion and fission events [19] and ER-endosome contact sites are important in controlling the association of endosomes with the cytoskeleton. ORP1L forms a complex at late endosomes with Rab7 and the Rab7 effector RILP. The p150^Glued^ subunit of the dynein/dynactin microtubule motor complex directly binds RILP, mediating minus-end-directed transport towards the nucleus. RILP additionally binds the homotypic fusion and vacuole protein sorting (HOPS) complex, mediating endosome tethering for fusion events [20]. Under conditions of low cholesterol in the endocytic pathway, ORP1L–VAPA interactions at ER-endosome contacts result in VAPA-mediated dissociation of RILP from p150^Glued^, with which VAPA associates in vitro, terminating its interaction with RILP [3]. RILP's interaction with the HOPS complex is also disrupted by ORP1L–VAP interaction [20], thus MCSs couple the regulation of both minus-end-directed transport with HOPS-mediated tethering and fusion (Fig. 2).

By terminating minus-ended transport, ORP1L–VAP interactions favour endosome transport towards the cell periphery. In a parallel, and perhaps coordinated mechanism, protrudin interaction with PI3P and Rab7 promotes the formation of contact sites that provide platforms for Kinesin-1 loading onto late endosomes. Kinesin-1 interacts with endosomal FYCO1 in a Rab7-dependent manner, promoting plus-end directed transport along microtubules (Fig. 2). As a consequence, endosomes are translocated towards the periphery, where synaptotagminVII-mediated fusion with the plasma membrane likely provides necessary lipids for the protrusion and neurite outgrowth that result [11].

Thus ER-endosome membrane contact sites coordinate the regulation of endosome motility and positioning with endosome tethering and fusion. Taken together with the demonstration that ER-endosome contacts define sites of endosome fission, these data reveal the importance of ER-endosome contact sites in the regulation of the complex functioning and maturation of the endocytic pathway. The role of cholesterol in this process is interesting. Under conditions of high cholesterol in the endocytic pathway, for example in cells from NPC patients, endocytic organelles accumulate in a perinuclear cluster [21]. Perinuclear clustering in cells lacking NPC1 was corrected by expression an ORP1L ΔORD mutant, lacking the oxysterol binding domain, resulting in scattered endosomes as the mutant ORP1L promotes interaction with VAPA at MCSs, disrupting the RILP/p150^Glued^ interaction and thereby terminating minus-end directed transport [3]. Rab7 binding of ORP1L and RILP thereby connects cholesterol sensing with endosomal positioning, such that under conditions of high cholesterol, endosomes are transported to the perinuclear region, presumably to facilitate fusion with predominantly perinuclear lysosomes.

Conversely, consistent with a role for ORP1L in MCS formation, contact sites were found to be increased under conditions of low cholesterol, with a consequent increase in a peripheral pool of endosomes [3]. Under these conditions, ORP1L adopts a conformation that interacts with VAPA at MCSs. Coincident interaction of Rab7 with ORP1L and RILP places RILP at the MCS, resulting in VAPA-mediated dissociation of RILP and dynein, and a consequent loss of perinuclear endosome transport. It is conceivable that endosome positioning might form part of the cell's response to low exogenous cholesterol. When exogenous cholesterol in the endocytic pathway is low, a feedback mechanism results in upregulation of the low density lipoprotein (LDL) receptor (LDLR) at the plasma membrane [22]. The increase in peripheral endosomes under these conditions might increase the efficiency of fusion of clathrin-coated vesicles rich in upregulated LDLR with early endosomes and sorting of the receptor back to the plasma membrane. Alternatively their peripheral proximity might facilitate fusion of recycling endosomes with the plasma membrane, possibly in a VAMP8-dependent manner, as has been demonstrated in cytotoxic T lymphocytes [23], or by synaptotagminVII-mediated endosome-plasma membrane fusion [11] to return essential membrane proteins and lipids to the plasma membrane for endocytosis of upregulated LDL receptor.

### 3.2 Regulation of growth factor receptors

Signalling from activated receptor tyrosine kinases (RTKs) is a key regulator of critical cellular processes, including proliferation, differentiation, survival and migration. As such, RTK activity must be tightly controlled and endocytic trafficking is central to the spatio-temporal regulation of RTK activity. Signalling from the EGF receptor tyrosine kinase, widely considered the prototypic RTK, occurs predominantly at the plasma membrane [24], but can also continue from the limiting membrane of endosomes where specific signalling pathways are activated [25].

Disregulated EGF receptor (EGFR) traffic can result in aberrant activity of its RTK and is associated with increased mitogenic signalling and tumourigenesis [26]. Thus regulation of RTK activity by endocytic trafficking has important consequences for cell fate. Activated EGFR is targeted for lysosomal degradation by ubiquitination, which mediates its interaction with components of the endosomal sorting complex required for transport (ESCRT) machinery and sorting onto endosomal intraluminal vesicles (ILVs) [27]. The ESCRT-dependent formation of ILVs within the lumen of the endosome, by a process of inward vesiculation from the limiting membrane, generates endosomes known as multivesicular bodies (MVBs). Sequestration of EGFR onto ILVs results in attenuation of signal as the active RTK is physically separated from its cytosolic targets. EGFR trafficking and endosome biogenesis has been extensively studied over many decades, but only relatively recently did the role of membrane contact sites in the spatial regulation of RTK activity become apparent.

The finding that an ER-localized phosphatase, protein tyrosine phosphatase 1B (PTP1B) interacts with endocytosed EGFR [28] sparked interest in how these two proteins might come into contact. Some ten years later, ER-endosome contact sites were identified that provide platforms for PTP1B-mediated effects at the endosome [2] (Fig. 2). PTP1B was found to directly dephosphorylate both EGFR and granulocyte colony stimulating factor receptors (G-CSFRs) at MCSs. G-CSFR on early endosomes also interacts with the ER-localized antioxidant peroxiredoxin Prdx4 and both G-CSFR and Prdx4 were found to be hyperphosphorylated in the absence of PTP1B [29]. PTP1B-mediated dephosphorylation of Prdx4 is predicted to increase its antioxidant activity, thereby maintaining PTP1B in an active state, with a resulting reduction in G-CSFR tyrosine phosphorylation. In addition, PTP1B activity promotes both dephosphorylation of the ESCRT components Hrs [2] and STAM [30] and, likely as a consequence of its ability to modulate the ESCRT machinery, the formation of ILVs. Thus PTP1B activity at ER-endosome contact sites mediates RTK dephosphorylation and sequestration onto ILVs, with a consequent downregulation of RTK activity. Paradoxically, while the predicted increase in ligand-stimulated phosphorylation of the EGFR was observed in PTP1B-null mouse fibroblasts [31], this was not accompanied by cell proliferation. This is possibly due to the increased tyrosine phosphorylation of p62Dok observed in PTP1B-deficient cells, resulting in decreased Ras activity [32]. Moreover, PTP1B activity was shown to promote ErbB2-dependent mammary tumorigenesis in vivo [33], [34], possibly by dephosphorylation of an inhibitory tyrosine residue in c-Src, resulting in Src activation [35].

## 4 Proposed functions for ER-endosome contact sites

### 4.1 Lipid transport

Lipid transport has been demonstrated across ER membrane contact sites with other organelles, including OSBP-mediated ER to Golgi sterol transfer [36]. That ORP1L, an OSBP homologue, is implicated in ER-endosome contact site formation through interaction with VAP on the ER, coupled with the regulation of ER-endosome contact site extent by cholesterol sensing [3], has led to speculation that these contact sites might also function in sterol transport [37], [38]. The majority of the cell's exogenous cholesterol is derived from LDL endocytosed via the LDL receptor, with the endocytic pathway reported to contain a significant amount of the total cellular cholesterol [39]. Hydrolysis of endocytosed LDL releases free cholesterol in endocytic organelles, which is transferred from the soluble NPC protein NPC2, in the lumen of the organelles, to membrane-bound NPC1 on the limiting membrane [40]. The ER-resident enzyme, Acyl-CoA:cholesterol acyltransferase (ACAT), catalyses cholesterol esterification prior to storage in lipid droplets [41]. However, the exact mechanism by which cholesterol reaches the ER from endocytic organelles remains unclear. The majority is believed to be transported first to the plasma membrane [42] and it is possible that MCSs might play a role in directing positioning/fission of endocytic vesicles for delivery to the plasma membrane.

The importance of NPC proteins for effective efflux of cholesterol from the endocytic pathway is well documented, with loss of NPC1 or NPC2 resulting in the accumulation of intracellular free cholesterol and sphingolipids [43]. It is estimated that in addition to indirect cholesterol transport via the plasma membrane, at least 30% of LDL-cholesterol reaches the ER by a direct, non-vesicular route [44], [45]. NPC1 on late endosomes interacts with the ER-resident oxysterol-binding protein-related protein 5 (ORP5), loss of which also causes accumulation of LDL-cholesterol in the endocytic pathway [46]. Thus the concept of direct transfer of LDL-derived cholesterol between NPC1 and ORP5 at ER-endosome contact sites has been mooted, but neither this, nor a direct role for NPC1 or ORP5 at contact sites has yet been demonstrated.

ORP5 also interacts with the ESCRT protein Hrs and loss of Hrs, like NPC1, also results in an accumulation of LDL-cholesterol in the endocytic pathway [47]. It is unclear why LDL-cholesterol egress should be disrupted by Hrs silencing. Since depletion of Tsg101, another component of the ESCRT machinery required for cargo sorting and endosome maturation, did not affect cholesterol transport [47], it is unlikely to be due to the loss of ESCRT-mediated sorting/maturation events. It is possible that Hrs is required for sorting sterol transfer proteins to cholesterol-rich microdomains on the limiting membrane, or that the stability of ORP5 interactions at the endosome is reduced in the absence of Hrs, with a consequent loss of lipid transfer. ORP5 was recently found to mediate PI4P/phosphatidylserine exchange at ER-plasma membrane contact sites [48], which could indicate a preference for phospholipid, rather than sterol binding. Alternatively, could ORP5 function in sterol/phosphoinositide exchange at ER-endosome contacts, as has been demonstrated for OSBP at ER-Golgi contact sites [36]?

How ORP1L might mediate the efflux of LDL-cholesterol from endocytic organelles is hard to rationalize since ORP1L-VAP interactions occur under conditions of low cholesterol in the endocytic pathway. More likely, sterol binding by ORP1L contributes to sterol sensing at the endosome, directing cholesterol-laden endosomes towards the peri-nuclear compartment for lysosome fusion. Since ORP1L can bind phosphoinositides through its PH domain, ORP1L might, like OSBP^36^, also mediate sterol/phosphoinositide exchange at ER-endosome contacts. Sterol transport across ER-endosome contact sites will no doubt be the subject of intense scrutiny in future studies.

### 4.2 Calcium signalling

Ca^2 +^ signals act as second messengers, mediating essential and wide-ranging physiological processes and are therefore subject to tight regulation. Cells achieve this by rapid spatiotemporal responses that result in localized increases in Ca^2 +^ concentrations by influx of extracellular Ca^2 +^ through Ca^2 +^ channels in the plasma membrane and release from intracellular Ca^2 +^ stores. The ER is the cell's major store of Ca^2 +^, which can be released through the ER's calcium channels inositol triphosphate (IP3) receptors or ryanodine receptors [49]. With Ca^2 +^ levels (approximately 0.5 mM), comparable with those found in the ER [50], endocytic organelles also represent a major intracellular Ca^2 +^ store and localized Ca^2 +^ signals are important in endosomal fusion events [51].

Functional coupling of these two major intracellular Ca^2 +^ stores at ER-lysosome contact sites has been proposed. Indeed direct mobilization of lysosomal Ca^2 +^ by osmotic permeabilisation is sufficient to evoke Ca^2 +^ release from the ER [52]. Furthermore, this coupling was also evident in the opposite direction. A study using sea urchin eggs, found that Ca^2 +^ release from the ER induced alkalinisation of endocytic organelles, that accompanies Ca^2 +^ release [53]. Computational modelling also supports the notion of bi-directional amplification of ER and endocytic organelle Ca^2 +^ signals at MCSs [54], [55]. It is known that Ca^2 +^ signals are required for normal endo-lysosomal fusion events [51] and that MCSs also play a role in regulating endosomal positioning and fusion events, as described above. Do Ca^2 +^ signals recruit tethering proteins to stabilize the contacts? ER-endosome contact sites were found to be increased (likely reflecting more stabilized contacts) following stimulation with EGF [2], which induces an increase in cytosolic Ca^2 +^ [56]. The consequent increased PTP1B-EGFR interaction likely stabilizes the MCS, but it is also possible that the Ca^2 +^ spike following EGF stimulation might serve to recruit a Ca^2 +^-sensitive tether to promote the formation of the ER-endosome contacts that provide sites of interaction for PTP1B with endocytosed EGFR.

Ca^2 +^ signals are important at ER contact sites with other organelles, including the plasma membrane and mitochondria and at these contacts there appears to be a close relationship between Ca^2 +^ signals and lipid transport. Data from NPC1 cells suggests that this might also be true at ER contacts with endocytic organelles. The accumulation of cholesterol in the endocytic pathway that characterizes NPC1 cells can be reproduced by chelating endocytic organelle Ca^2 +^ stores. Moreover, Ca^2 +^ levels in endocytic organelles are reduced in NPC1 cells and inducing Ca^2 +^ release by the ER corrects the cholesterol accumulation in these cells [50]. It is possible that Ca^2 +^ signals might be required to promote not only endo-lysosome fusion but also ER contact sites for the efflux of LDL-cholesterol from endocytic organelles. Could elevations in Ca^2 +^ at contact sites recruit tethering proteins required to stabilize and extend the contact, providing a platform for sterol transfer from the endosome? The sterol transfer proteins that compensate for the lack of NPC1 on Ca^2 +^ release from the ER remain elusive, but ORP1L is a likely candidate, since expression of an adenoviral membrane protein, RIDα, rescues the cholesterol accumulation phenotype in NPC1 cells in an ORP1L-dependent manner, promoting lipid droplet formation [57]. How ORP1L transfers sterols under conditions of high LDL-cholesterol is not yet fully understood, but ORP1L's interaction with the Oas1b-containing complex in the ER [58] could be involved. The observation that ORP5 on the ER is able to bind endosomal phospholipids through its PH domain [59], raises the possibility that a sterol transfer protein at the endosome might not be required, but rather ORP5 might retrieve sterols from microdomains on the limiting membrane of the endosome.

The bidirectional amplification of ER and lysosome Ca^2 +^ signals is widely believed to occur at contact sites between the two organelles. It is likely that these localized elevations in Ca^2 +^ signals promote endosome–lysosome fusion, consistent with the key role for MCSs in regulating the maturation and functioning of the endocytic pathway. Whether or not Ca^2 +^ also serves to promote the formation of contact sites between the ER and endocytic organelles will surely become apparent in the near future.

## 5 Conclusions and perspectives

Just as there are multiple populations of endocytic organelles [13], [60], it seems likely that multiple populations of ER contact sites with endocytic organelles also exist, with organelle-specific tethering complexes, that might for example, be recruited by Rab5 or Rab7, markers of early or late endosomes respectively.

Several interactions between endosome and ER-localized proteins have been reported, that may, as discussed, stabilize existing MCSs, but no single regulator of ER-endosome contacts has been identified to date. It is likely that, as is the case for ER-plasma membrane contact sites [61], multiple interactions occur at the MCS, such that loss of any individual component has little effect. Such a robust system of interorganellar communication is likely to reflect the physiological importance of the interactions and exchange that occur at contact sites. Indeed MCS disruption has been implicated in a number of diseases [37]. The yeast protein Mdm1 was recently identified as a tethering component for ER-vacuole contact sites [62]. The role of the mammalian Mdm1 homologue, sorting nexin 14 (SNX14), in tethering ER contact sites with endocytic organelles has not yet been established. However, Mdm1 truncations analogous with SNX14 mutations that are associated with a neurological disorder characterized by intellectual disability and cerebella atrophy, failed to tether ER-vacuole contact sites in yeast, strongly implicating ER contact sites with endocytic organelles in the pathogenesis of the disease associated with SNX14 mutations.

While our understanding of the biology of membrane contact sites between the ER and endocytic organelles has progressed enormously in recent years, much remains to be elucidated. Recent advances in super-resolution and 3D microscopy techniques should further accelerate progress in this rapidly developing field.

## Conflict of interest

I wish to confirm that there are no known conflicts of interest associated with this publication and there has been no significant financial support for this work that could have influenced its outcome.
